# Far-Ultraviolet C Disinfection Reduces Oxidative Damage to the Cornea Compared to Povidone-Iodine Disinfection

**DOI:** 10.3390/antiox13111344

**Published:** 2024-11-01

**Authors:** Tu-Wen Chen, Rong-Kung Tsai, Cheng-En Zou, Chin-Te Huang, Maisam Ali, Tzu-Chao Hsu, Keh-Liang Lin, Yao-Tseng Wen

**Affiliations:** 1 Institute of Eye Research, Hualien Tzu Chi Hospital, Buddhist Tzu Chi Medical Foundation, Hualien 970473, Taiwan; qooqoo700@tzuchi.com.tw (T.-W.C.); rktsai@tzuchi.com.tw (R.-K.T.); a50502468@gmail.com (C.-E.Z.); 110726111@gms.tcu.edu.tw (M.A.); 2 Institute of Medical Sciences, Tzu Chi University, Hualien 970374, Taiwan; 3 Doctoral Degree Program in Translational Medicine, Tzu Chi University and Academia Sinica, Hualien 970374, Taiwan; 4 Department of Ophthalmology, School of Medicine, Chung Shan Medical University Hospital, Chung Shan Medical University, Taichung 402306, Taiwan; 5 Medical Administration Office, Department of Medical Education, Hualien Tzu Chi Hospital, Buddhist Tzu Chi Medical Foundation, Hualien 970374, Taiwan; ms0374577@gmail.com; 6 Department of Optometry, Mackay Medical College, New Taipei City 252005, Taiwan; eye490507@gmail.com

**Keywords:** 222 nm far-UVC, cornea, disinfection, povidone-iodine, oxidative damage, apoptosis safety

## Abstract

Far-ultraviolet C (far-UVC) light shows promise for pathogen control but its safety and efficacy for corneal disinfection remain unclear. In this study, safe far-UVC dosages were investigated for corneal disinfection and its germicidal performance and oxidative damage potential to 5% povidone-iodine (PVP-I) were compared. Rat corneas were exposed to varying 222 nm far-UVC doses (3–60 mJ/cm^2^) and assessed for ocular damage, apoptosis, and oxidative stress to determine the safe dose of far-UVC. Far-UVC at 30 mJ/cm^2^ induced corneal apoptosis and oxidative damage, but 15 mJ/cm^2^ caused no apoptosis or oxidative damage. At this optimized dose (9 mJ/cm^2^), far-UVC achieved 90.5% sterilization, exceeding 5% PVP-I (80.8%), with significantly less oxidative damage and cell death in the cornea. In conclusion, our study demonstrates that the use of 5% povidone-iodine (PVP-I) for disinfection results in significant oxidative damage to the corneal tissue. However, a safe dosage of far-UVC light exhibited a promising disinfection effect without causing oxidative damage to the corneal tissue. Far-UVC offers a promising alternative for corneal disinfection but requires careful dosage control (≤30 mJ/cm^2^) to avoid ocular surface harm.

## 1. Introduction

The microbiome of the eye, encompassing the microflora of bacteria, fungi, and viruses detected on the conjunctiva and cornea, plays a vital role in maintaining ocular health. A healthy ocular surface microbiome is characterized by relative stability, safety, and low diversity, contributing to homeostasis and immune response modulation. However, an unhealthy or injured ocular surface, particularly following surgery, can become susceptible to microbial penetration of the corneal epithelium, potentially leading to various infectious eye diseases. *Pseudomonas, Staphylococcus*, and *Streptococcus* species are among the most prevalent bacterial pathogens implicated in corneal infections [1,2]. To mitigate these serious complications, surgeons often employ 5% povidone-iodine (PVP-I) and administer pre- and postoperative antibiotics [3,4].

While a universally accepted regimen for infection prophylaxis remains elusive, PVP-I is frequently utilized for ocular surface disinfection prior to ophthalmic surgery, aiming to prevent endophthalmitis [5]. This practice is attributed to PVP-I’s broad-spectrum antibacterial activity, low propensity for inducing bacterial resistance [6], and efficacy against fungi and viruses [5]. Diiodine, or “free iodine,” a component of PVP-I, readily penetrates bacterial membranes via porins, causing protein oxidation in the bacterial cytoplasm [7]. A 5–10% PVP-I solution is recommended for conjunctival surface disinfection, offering advantages such as low cost, wide availability, and non-toxicity to the eyes at a 5% concentration [8,9].

Despite its effectiveness in ocular surface disinfection, PVP-I can induce eye irritation or allergic reactions, manifesting as gritty or tingling sensations and redness [10]. This is due to the continuous release of iodine onto the ocular surface following application. A recent study showed that excessive iodine levels lead to cell growth inhibition, oxidative stress, and cellular apoptosis in pancreatic beta cells [11]. In addition, clinical and experimental data have demonstrated the toxic effects of topical iodine on the ocular surface epithelium, especially with repeated exposure [12]. Prolonged use of 5% PVI can lead to keratocyte death and persistent epithelial defects in the damaged corneal epithelium. This occurs due to the disruption of the stromal–epithelial interaction, which results in delayed re-epithelialization [13]. However, there is limited understanding of the oxidative damage to the cornea resulting from the use of 5% povidone-iodine (PVI) for ocular surface disinfection.

Ultraviolet (UV) light, particularly the UVC band (100–280 nm) known as ‘germicidal UV’, presents an effective and direct antimicrobial disinfection method [14]. The 254 nm wavelength is commonly employed for germicidal purposes, but prolonged direct exposure can be detrimental to bodily surfaces [15]. In contrast, far-UVC light (207–222 nm) has demonstrated comparable efficacy to 254 nm UV light while posing less risk to human tissues [16]. Recent studies have substantiated the disinfection capacity and safety of 222 nm far-UVC light on skin tissue, highlighting its potential in animal models [17]. The shallow penetration depth of far-UVC contributes to its safety profile, making it suitable for surface cleaning, disinfection, and potentially early infection control in indoor environments. Unlike skin, which is shielded by a UV-absorbent keratinized squamous layer, the corneal epithelium is protected by a tear film which has difficulty absorbing most UV exposure. [18]. This necessitates careful evaluation of safe 222 nm far-UVC dosages for corneal exposure, particularly in indoor settings.

In this study, the aim was to investigate safe far-UVC exposure levels for rat corneal tissue and optimize the germicidal efficacy of these safe doses on the rat ocular surface. Additionally, a comparison was conducted between 222 nm far-UVC and 5% PVP-I to assess the relative effectiveness of far-UVC for ocular surface disinfection.

In this research, the potential is explored of far-UVC for corneal disinfection, emphasizing the importance of establishing safe dosage parameters. Far-UVC demonstrates promise as an alternative to traditional PVP-I, offering comparable or superior germicidal efficacy with reduced adverse effects. However, careful dosage control is imperative to ensure the safety of the ocular surface when utilizing far-UVC for disinfection purposes.

## 2. Materials and Methods

### 2.1. Study Design

A KrCl excimer lamp-equipped far-UVC lamp (Care222^®^; DELTA ELECTRONICS INC, Taipei, Taiwan) with a narrowband spectrum (200–230 nm, peak at 222 nm) was utilized for corneal surface disinfection. All treatments were performed after anesthetization. In the first stage, to determine safe far-UVC dosages, 27 rats were divided into nine groups (0, 3, 6, 9, 12, 15, 30, 45, and 60 mJ/cm^2^), and the following exposure times of 222 nm far-UVC were 0, 1, 2, 3, 4, 5, 10, 15, and 20 s, individually. According to a study by Wickert et al. [19], rat eyes were exposed to UV radiation to explore tissue proto-oncogenes and cell death. Then, 6 h after UV treatment, the eyes were removed and were frozen in sections for subsequent staining analysis. Intense labeling of DNA breaks was observed in numerous cells in the uppermost layers of the corneal epithelium by terminal deoxynucleotidyl transferase dUTP nick end labeling (TUNEL) assay. Thus, anesthetized rats received far-UVC exposure, and after 6 h, eyeballs were harvested for corneal section preparation [19]. Hematoxylin and eosin (H&E) staining was used to observe whether 222 nm far-UVC irradiation caused corneal damage. TUNEL assay for apoptotic cells and dihydroethidium (DHE) stain for reactive oxygen species (ROS) levels were assessed in the corneal sections.

In the second stage, viable bacteria on the rat ocular surface were quantified to optimize germicidal efficacy within the safe exposure window (3–15 mJ/cm^2^). A total of 18 rats were divided into six groups (0, 3, 15, 30, 45, and 60 mJ/cm^2^), and the following exposure times of 222 nm far-UVC were 0, 1, 5, 10, 15, and 20 s, individually. After treatment, we applied a cotton swab with topical 0.5% Alcaine eye drops (Alcon, Puurs, Belgium) to collect the bacteria from the lower conjunctival fornix of the rat. See Section 2.4. for bacterial inoculation and identification steps. The total number of bacterial colonies was counted through ImageMaster 2D Platinum Software V 7.0 (GE Healthcare, Chicago, IL, USA), and the results showed that 222 nm far-UVC can disinfect rat corneas and provide optimized exposure time.

In ophthalmic surgery, studies have shown that 5% PVP-I is highly effective at killing bacteria on the ocular surface. In the last stage, to compare far-UVC with 5% PVP-I, rat ocular surfaces were exposed to either 9 mJ/cm^2^ far-UVC or 5% PVP-I for 3 min. A total of 9 rats were divided into three groups 0 mJ/cm^2^, 9 mJ/cm^2^, and 5% PVP-I. We use the sindine solution (No. 00662, Sinphar Group, Yilan, Taiwan) in clinical ophthalmic surgery. According to clinical usage, 5% PVP-I was prepared by mixing sindine solution with normal saline at a ratio of 1:1 by volume. The bacteria were from the lower conjunctival fornix of the rat, and eyeballs were harvested for the corneal section. The total bacterial colony count, apoptotic cells, and ROS levels in corneal sections in each group were analyzed to evaluate whether 222 nm far-UVC (9 mJ/cm^2^) had a better corneal disinfection effect than 5% PVP-I.

### 2.2. Animals

Adult male Wistar rats (150–180 g, 7–8 weeks old) were obtained from BioLASCO Co., Taipei, Taiwan. All procedures adhered to the ARVO Statement for the Use of Animals and were approved by the IACUC of Buddhist Tzu Chi Medical Center. The rats were anesthetized by intramuscular injections of a mixture of ketamine (100 mg/kg body weight) and xylazine (10 mg/kg body weight; Sigma, St. Louis, MO, USA), and topical 0.5% Alcaine was used for general anesthesia. Rats were housed in a controlled environment with free access to food and water.

### 2.3. Measurement of Far-UVC Dosage

With support from Delta Electronics, we determined the dosage of far-UVC. The UIT2400 m (Ushio America Inc., Cypress, CA, USA) was used to measure irradiance in units of mW/cm^2^ directly. According to the irradiance readings, Care222^®^ at a distance of 2 cm from the UIT2400 m generates 3 mW/cm^2^. Therefore, by adjusting the exposure time from 1 to 20 s, it is possible to accurately produce a far-UVC dosage ranging from 3 to 60 mJ/cm^2^.

### 2.4. Bacterial Inoculation and Identification

After treatment, we applied a cotton swab with topical 0.5% Alcaine eye drops (Alcon, Puurs, Belgium) to collect the bacterial samples from the lower conjunctival fornix of a rat. The bacteria were smeared and cultured on the Trypticase Soy Agar plates (TSA; BD Biosciences, BD236950, Meylan, France, bought from the Department of Laboratory Medicine, Hualien Tzu-Chi Hospital, Hualien, Taiwan), and bacterial samples were collected from each eye and smeared on one plate. The plates were sent to the Department of Laboratory Medicine, Hualien Tzu-Chi Hospital for bacterial identification. After incubation at 35 °C for 18–20 h, a single colony was picked and homogenized in 300 μL ddH_2_O, add 900 μL 99.5% alcohol, and mix evenly after voting. The dissolved colony was centrifuged at 15,000 rpm for 2 min, and the supernatant was removed. The centrifugation step was repeated once, and the sample was air-dried at room temperature for 2 min. Then, 10 μL of 70% formic acid was added, and the mixture was vortexed. After that, 10 μL of ACN was added, mixed thoroughly, and centrifuged at 15,000 rpm for 2 min. A 1 μL aliquot of the supernatant was taken, placed on a MALDI plate, and air-dried at room temperature. Then, 1 μL of HCCA was added, placed on the MALDI plate, air-dried at room temperature, and the sample was introduced into MALDI-TOF for strain identification. The total colony numbers were counted using ImageMaster 2D Platinum Software V 7.0 (GE Healthcare, Chicago, IL, USA).

### 2.5. Preparation of Corneal Sections

The rat eyeballs were harvested after euthanizing the rats. A solution of 4% buffered paraformaldehyde was used to fix the samples. The eyeballs were then dehydrated in 30% sucrose. A plastic mold was then used to embed the eyeballs in optimal cutting temperature (OCT) compounds (Tissue-Tek^®^ O.C.T. Compound, SKU 4583; Sakura, Torrance, CA, USA). Liquid nitrogen was then used to freeze the molds completely. After transferring the frozen bricks to cryostats at −20 °C, 15 µm of the thickness of sections was collected. Sections were stored at −20 °C for further analyses.

### 2.6. Hematoxylin and Eosin (H&E) Stain

For staining, sections were dipped in gradient ethanol, stained with hematoxylin for 1 min, rinsed in 1X PBS for 5 min, and stained with eosin for 10–15 s. Several rinses in varying alcohol percentages at 75%, 95%, and 99% for two minutes were performed sequentially. After dehydrating with 99% ethanol, the slides were cleared twice with xylene for 2 min each. Observations were conducted under light microscopy after slides were mounted on neutral gum and dried.

### 2.7. TUNEL Assay for Apoptosis

As a result of fixation with 4% paraformaldehyde (PFA) and cryoprotection with 30% sucrose, frozen sections of the cornea were prepared with samples cut near the central cornea to ensure the comparison was accurate. After washing the microscope slides three times with 1X phosphate-buffered saline (PBS) for 5 min each, the borders of the sample were marked with a liquid blocker. This was performed to restrain the reagents during the procedure. Using Proteinase K solution (20 μg/mL, proteinase K in 0.3% Triton X-100, 1X PBS), we incubated the corneas for 10 min to increase permeability. At room temperature, the samples were incubated for 10 min in the equilibrium buffer provided by the kit. To detect apoptotic cells, we followed the protocol provided by the manufacturer (DeadEnd™ Fluorometric TUNEL System; Promega Corporation, Madison, WI, USA) [20]. The samples were washed with PBS for 10 min each, and then the nuclei (blue) were stained with 4′, 6-diamidino-2-phenylindole (DAPI, 1:100; Sigma, St Louis, MO, USA). Images were obtained from the central and mid-peripheral cornea using a confocal microscope. TUNEL-positive cells (green) were counted in the corneal epithelial layer of each sample in ten high-powered fields (HPFs, 400× magnification) selected randomly [21]. The average was then calculated for further comparison.

### 2.8. Quantification of ROS Production

The levels of ROS in the cornea were quantified by dihydroethidium (DHE; Thermo Fisher Scientific, Waltham, MA, USA), which gives bright fluorescent red when oxidized to ethidium in the presence of ROS [22]. The sections were prepared from samples cut at 10–20 µm thickness in an optimal cutting temperature (OCT) compound (Tissue-Tek^®^ O.C.T. Compound, SKU 4583; Sakura, USA). Following gentle washing in PBS and blocking with 3% fetal bovine serum (FBS) at room temperature for 1 h, frozen cross sections were incubated with 100 µM DHE for 30 min at 37 °C. The sections were washed in PBS once to stain the nuclei and mounted in 4′, 6-diamidino-2-phenylindole (DAPI, 1:100; Sigma, St Louis, MO, USA). The images were taken with a fluorescence microscope.

### 2.9. Statistical Analysis

Data were analyzed using GraphPad Prism software version 8.0 (San Diego, CA, USA). Results are presented as means ± SD. The Kruskal–Wallis test with post hoc tests was used for analysis. Statistical significance was set at *p* < 0.05.

## 3. Results

### 3.1. The Exposure Limit of Far-UVC on Rat Cornea Is 15 mJ/cm^2^ to Avoid Corneal Damage

**Figure 1:**
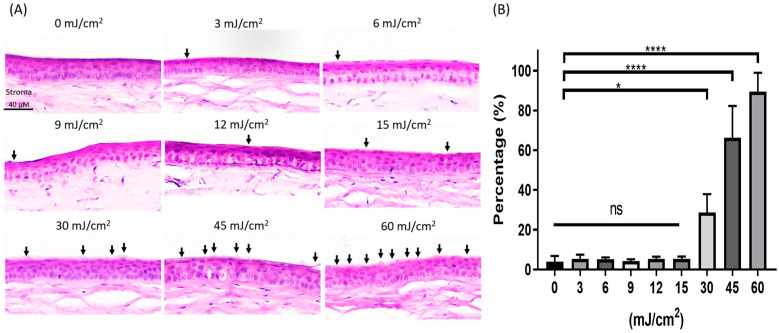
Analysis of the corneal epithelium damage through the H&E stain after far-UVC exposure. (**A**) Representative images of the corneal section in the different far-UVC dose-treated groups. Arrows point to ruptured areas or lysed cells of the corneal epithelium. (**B**) Quantification of ruptured areas or lysed cells in the corneal epithelium for each group. (* *p* < 0.05, **** *p* < 0.0001). Data are expressed as the mean ± SD.; H&E, hematoxylin, and eosin staining. ns, not significant. Scale bar: 40 μm.

The cornea was examined using H&E staining to evaluate the structural changes in the corneal tissue (Figure 1A). The proportion of corneal damage per 100 µm of corneal length was used to calculate the amount of the injury. Data were analyzed using GraphPad Prism. The statistical analyses were presented as means ± SD. A non-parametric *t*-test (Mann–Whitney U-test) was employed to compare differences among groups. Results with *p*-values less than 0.05 were statistically significant. Far-UVC exposure of 3 mJ/cm^2^ to 15 mJ/cm^2^ showed no statistical difference in the corneal epithelium rupture or lysis level compared to the unexposed cornea (Figure 1B; *p* > 0.9999 vs. 0 mJ/cm^2^). Far-UVC exposure of 30, 45, and 60 mJ/cm^2^ resulted in a higher corneal epithelium rupture or lysis than the unexposed cornea. (Figure 1B; *p* < 0.05 vs. 0 mJ/cm^2^).

### 3.2. The Exposure Limit of Far-UVC on Rat Cornea Is 15 mJ/cm^2^ to Prevent Oxidative Damage and Epithelium Apoptosis in the Cornea

**Figure 2:**
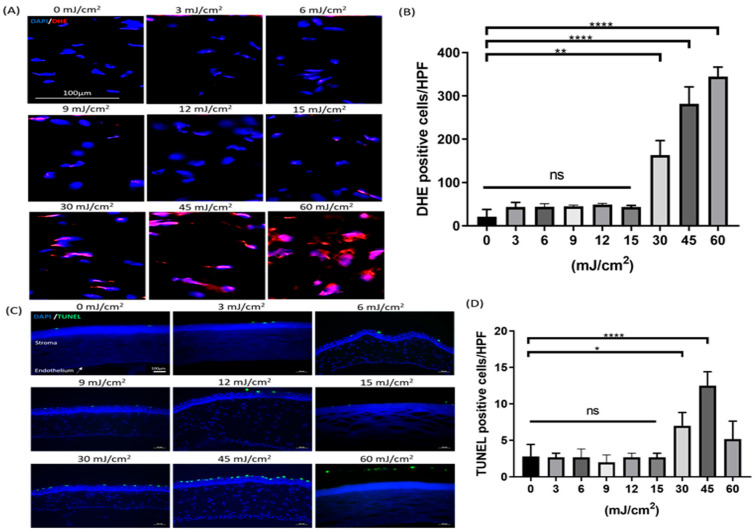
The corneal injuries after the designated doses of far-UVC exposure. (**A**) Representative images of fluorescent staining of oxidative stress in the corneal stroma. (**B**) Quantification of the dihydroethidium (DHE)-positive cells per high-power field (HPF). The number of DHE-positive cells in the 30, 45, and 60 mJ/cm^2^ of far-UVC-treated groups were significantly higher than the non-treated group (** *p* < 0.01, **** *p* < 0.0001). (**C**) Representative images of apoptotic cells in the corneal epithelium. (**D**) Quantification of the number of the TUNEL-positive cells per HPF. The number of apoptotic cells in the 30 and 45 mJ/cm^2^ of far-UVC-treated groups was significantly higher than in the non-treated group (* *p* < 0.05, **** *p* < 0.0001). Data are expressed as the mean ± SD. DHE, dihydroethidium; HPF, high-power field; TUNEL, TdT-dUTP nick end-labeling. ns, not significant. Scale bar: 100 μm.

No visible DHE-labeled cell was detected in the corneal stroma layer in the 0, 3, 6, 9, 12, and 15 mJ/cm^2^ of far-UVC-treated groups (Figure 2A). It indicated that far-UVC exposure of 3 mJ/cm^2^ to 15 mJ/cm^2^ did not induce more oxidative stress in the corneal stroma cell compared to the unexposed cornea (Figure 2B; *p* > 0.9999 vs. 0 mJ/cm^2^). However, far-UVC exposure of 30, 45, and 60 mJ/cm^2^ induced a higher level of DHE-positive cells in the corneal stroma layer compared to the unexposed cornea (Figure 2B; *p* < 0.05 vs. 0 mJ/cm^2^). Thus, far-UVC exposure over 30 mJ/cm^2^ resulted in high production of ROS in the corneal stroma cells. No significant differences were observed in the number of TUNEL-positive cells among the 0, 3, 6, 9, 12, and 15 mJ/cm^2^ of far-UVC-treated groups (Figure 2C; *p* > 0.9999). Nevertheless, far-UVC exposure of 30 and 45 mJ/cm^2^ induced a higher number of the TUNEL-positive cell on the first layer of corneal epithelium compared to the unexposed cornea (Figure 2D; *p* <0.05 vs. 0 mJ/cm^2^). The highest dose (60 mJ/cm^2^) of far-UVC did not induce more apoptosis on corneal epithelium compared to unexposed eyes (Figure 2D; *p* = 0.3294) because the outermost corneal epithelial cells were destroyed and detached on the corneal epithelium after far-UVC exposure. Thus, the number of apoptotic cells on the corneal section was not stable to be stained.

### 3.3. Nine mJ/cm^2^ Is the Optimal Dose of Far-UVC for Safe Disinfection on the Ocular Surface

**Figure 3:**
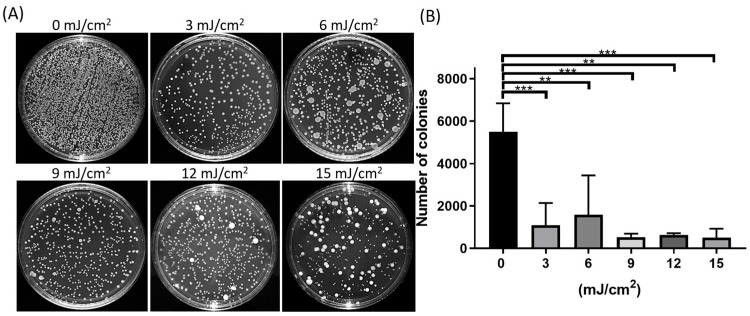
The total viable count on rat ocular surface after far-UVC exposure. (**A**) Representative images of total viable count for rat ocular surface after far-UVC exposure. (**B**) Quantification of total colonies for each group. The CFU in the far-UVC-treated groups was significantly lower than in the non-treated group (** *p* < 0.01, *** *p* < 0.001, *n* = 4 per group). Data are expressed as the mean ± SD.

The safety assessment was based on H&E, DHE, and TUNEL assays (Figure 1 and Figure 2); the safe exposure dose of far-UVC should be less than 15 mJ/cm^2^ to prevent corneal injuries. We used 3, 6, 9, 12, and 15 mJ/cm^2^ of far-UVC to disinfect the ocular surface. Subsequently, the total viable count was performed on the rat ocular surface (Figure 3A). The viable bacteria were significantly decreased in the far-UVC-treated groups compared with the non-treated group (Figure 3B; *p* < 0.001 vs. 0 mJ/cm^2^). The 3 and 6 mJ/cm^2^ of far-UVC-treated groups showed higher standard deviations than other treated groups. Thus, we determined that 9 mJ/cm^2^ of far-UVC is the optimal dose for safe disinfection on the ocular surface.

### 3.4. Disinfection Efficacy of the Optimal Dose of Far-UVC Is Similar to 5% PVP-I Treatment

**Figure 4:**
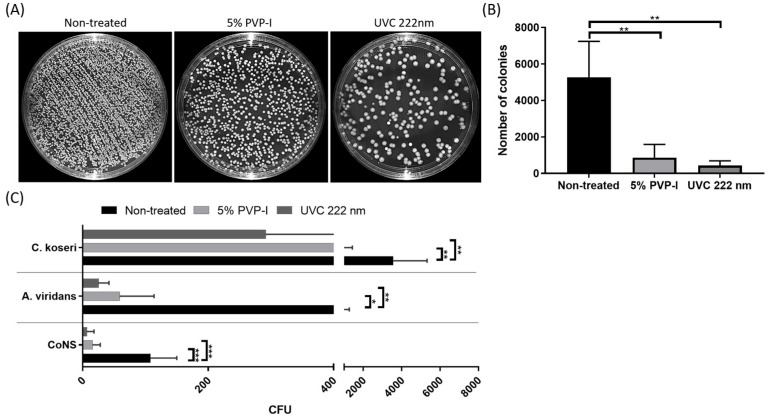
Comparison of disinfection efficacy between 5% PVP-I and 9 mJ/cm^2^ of far-UVC in rat ocular surface. (**A**) Representative images of the total colonies on the TSA plate for each group. (**B**) Quantification of total colonies on the TSA plate. The number of the total colonies in the 5% PVP-I-treated and 9 mJ/cm^2^ of far-UVC-treated groups was significantly lower than in the non-treated group (** *p* < 0.01). (**C**) Quantification of the colony number of 4 bacterial species. The lowest colony number was found in the 9 mJ/cm^2^ of the far-UVC-treated group for all bacterial species (* *p* < 0.05, ** *p* < 0.01, *** *p* < 0.001). Data are expressed as the mean ± SD.

The total number of colonies in the 5% PVP-I- and 9 mJ/cm^2^ of far-UVC-treated groups were significantly decreased by 80.8% and 90.5% compared to those in the non-treated group (Figure 4A,B; *p* < 0.001 vs. 0 mJ/cm^2^). However, there was no significant difference in CFU count between the 5% PVP-I-treated group and the 9 mJ/cm^2^ of the far-UVC-treated group (Figure 4A,B; *p* = 0.8525). In addition, four different bacterial species were identified on rat ocular surface, including coagulase-negative staphylococci (CoNS), *Aerococcus viridans* (*A. viridans*), and *Citrobacter koseri* (*C. koseri*) (Figure 4C). Total CFU counts of CoNS, *A. viridans*, and *C. koseri* were reduced in the 5% PVP-I-treated and the 9 mJ/cm^2^ of far-UVC-treated groups compared with the non-treated group (Figure 4C; *p* < 0.05 vs. 0 mJ/cm^2^).

### 3.5. Far-UVC Exposure of 9 mJ/cm^2^ on the Cornea Induced Lower Oxidative Stress and Epithelium Apoptosis than 5% PVP-I Treatment

**Figure 5:**
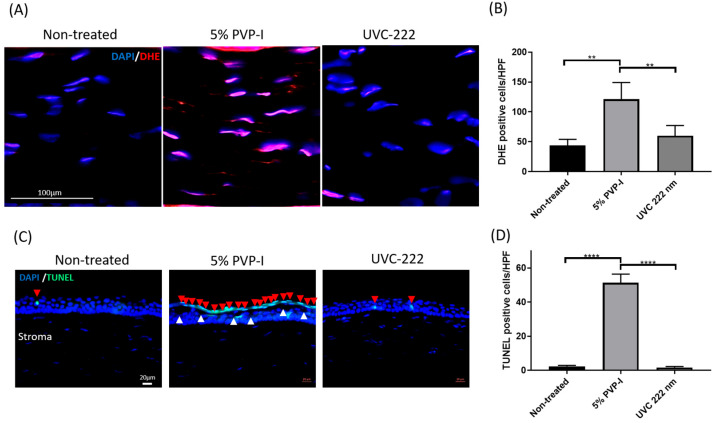
Comparison of corneal injuries in the rat cornea between 5% PVP-I treatment and 9 mJ/cm^2^ of far-UVC exposure (**A**) Representative images of the oxidative stress in the corneal stroma for each group. (**B**) Quantification of the DHE-positive cells per HPF. The number of DHE-positive cells in the 5% PVP-I-treated group was significantly higher than those in the non-treated group and the 9 mJ/cm^2^ of the far-UVC-treated group (** *p* < 0.01, *n* = 6 per group). (**C**) Representative images of apoptotic cells in the corneal epithelium in the non-treated, the 5% PVP-I-treated, and the 9 mJ/cm^2^ of the far-UVC-treated group. Red arrowheads indicate the TUNEL-positive cells, and white arrowheads indicate the cavities in the corneal epithelium. (**D**) Quantification of the number of TUNEL-positive cells per HPF. A significant increase in the number of TUNEL-positive cells was found in the 5% PVP-I-treated group than the other groups (**** *p* < 0.0001). Data are expressed as the mean ± SD. HPF, high-power field; dihydroethidium (DHE)-labeling. TUNEL, TdT-dUTP nick end-labeling. Scale bar: 100 μm.

There was no statistically significant difference in the number of DHE-positive cells between the non-treated group and the 9 mJ/cm^2^ of the far-UVC-treated group (Figure 5A,B; *p* = 0.3923). However, the number of DHE-positive cells in the 5% PVP-I-treated group was significantly higher than those in the non-treated group by 2.79-fold and the far-UVC-treated group by 1.40-fold (Figure 5B; ** *p* < 0.01). TUNEL assay demonstrated no statistical difference in the number of apoptotic cells between the non-treated group and the 9 mJ/cm^2^ of the far-UVC-treated group (Figure 5C,D; *p* = 0.3923). As for the 5% PVP-I-treated group, there was a significant increase in apoptotic cells of the corneal epithelium than the other groups (Figure 5C,D; *p* < 0001). Furthermore, the cavities (white arrows) within the corneal epithelium were only found in the 5% PVP-I-treated group. These cavities resembled ballooned vacuolated superficial epithelial cells. We found that 5% PVP-I had injured the corneal epithelium, harming the cornea’s outermost cells and making them susceptible to separation.

## 4. Discussion

In the eye, previous studies have suggested that acute UV exposure causes acute keratitis [23], while chronic UV exposure can result in pterygium [24], conjunctival tumors [25], and cataracts [26]. So far, recent investigations have proved that short-wave ultraviolet (far-UVC) has a bactericidal effect on viruses and bacteria [27], but is much less hazardous to higher organisms, including humans, than previously believed [28,29,30,31].

Rats were used in the recent studies to assess the corneal damage induced by UV rays of various wavelengths, including 222 nm far-UVC [29]. At 222 nm far-UVC, the threshold for radiation exposure ranges from 3500 to 5000 mJ/cm. Compared to longer UV wavelengths, these cells are far less damaging to the cornea and usually shed within 24 h of their natural turnover cycle. Kaidzu et al. [32] also confirmed that 222 nm far-UVC reached only the outermost layer of the rat’s corneal epithelium. Porcine corneas, comparable to human eyes in size and structure, showed the same outcomes as rats; only the superficial layer of the corneal limbal epithelium was exposed to 222 nm far-UVC. Only the outermost layer of the corneal epithelium is penetrated by 222 nm far-UVC. The minimum dose that meets the corneal damage caused by far-UVC is much higher than the threshold limit value (TLV^®^) promulgated by the American Conference of Governmental Industrial Hygienists (ACGIH) (The American Conference of Governmental Industrial Hygienists (2020) TLVs^®^ and BEIs ^®^ Cincinnati, OH, USA).

In this study, it was established that 15 mJ/cm^2^ of far-UVC disinfection does not damage the corneal tissue in rats. Previous reports have indicated the following varying thresholds for 222 nm UVC-induced corneal damage in other species: 45 mJ/cm^2^ in rabbits, 20 mJ/cm^2^ in primates, 10 mJ/cm^2^ in humans, and up to 600 mJ/cm^2^ in mice [29,33,34]. The observed interspecies differences in corneal susceptibility to 222 nm far-UVC might be attributed to variations in the assessment criteria for corneal injury or the inclusion of deeper corneal layers in some studies. Our findings confirm that 15 mJ/cm^2^ is a safe dose for rat corneas, inducing no oxidative damage to the epithelium or stroma.

Although the tear film partially absorbs far-UVC, over 80% can penetrate to the outermost corneal epithelium [35]. In our rat model, 30 mJ/cm^2^ of 222 nm far-UVC induced superficial punctate keratitis and some apoptotic cells in the superficial epithelial layer. Higher doses (45–60 mJ/cm^2^) increased corneal erosion. However, the rapid turnover of the corneal epithelium (5–7 days) suggests that affected surface cells are likely shed within 24 h. Therefore, the frequent use of high-dose far-UVC may bring the hazard risk for corneal disinfection. We refer to Ikuta et al. [36], who used cryosection samples stained with H&E to assess rat corneal histology. The water in the frozen section sample may create ice crystals if the freezing procedure is not completed quickly enough, which could seriously damage or deform the biological sample, according to Taylor et al. [37]. The sample’s microstructure will be destroyed by ice crystals, which will lower the imaging quality. In our images, damage to the outermost corneal epithelium with exposures exceeding 30 mJ/cm^2^ at 222 nm far-UVC can still be observed.

So far, various PVP-I concentrations ranging from 0.025% to 10% and incubation times ranging from seconds to minutes have been used worldwide [38,39]. Due to PVP-I’s negative effects on the eyes, particularly corneal toxicity, it is advised that the concentration, pH, and exposure duration be carefully regulated [40,41]. In ophthalmic surgery, 5% PVP-I is widely used as a preoperative antiseptic to reduce the risk of postoperative infections, such as endophthalmitis [42,43]. Most studies believe that 5% PVP-I is highly effective at killing bacteria on the ocular surface and is safe for the cornea. In this study, it is indicated that while past research has seldom explored whether 5% PVP-I in ocular disinfection may cause oxidative damage to the cornea, most studies have considered 5% PVP-I a safe method without leading to oxidative damage [38,44,45]. The results of this study demonstrate that the use of 5% PVP-I does indeed increase oxidative stress in corneal cells, further triggering apoptosis, which is inconsistent with the safety of 5% PVP-I in ophthalmic surgery for human clinical preoperative applications [42,43,46]. Previous studies have also mentioned that PVP-I could cause corneal cell death, primarily through cell fixation mechanisms, which differs from the mechanisms observed in this study [13]. Additionally, recent research has found that excessive iodine can cause oxidative damage to the pancreas in normal rats [11]. In this study, it was demonstrated that rats can develop oxidative stress and pancreatic damage after receiving 500 times the daily physiological dose of iodide for 60 days. It has been established that iodide can cause oxidative stress on the pancreas even after intestinal digestion. In our study, rats’ corneas were directly exposed to 5% PVP-I, which caused oxidative stress and the death of corneal epithelial cells. Although the tissues examined in that study differ from ours, it confirms that iodine can induce tissue oxidative damage.

Notably, antiseptic agents used during ophthalmic procedures may be pathogenic factors that harm the cornea and result in wound-healing disorders. Rabbits were studied for ocular surface damage brought on by exposure to 5% PVI in a time-dependent manner (incubation up to 10 min) [47]. Furthermore, in a rabbit model, epithelial injury was noted 30 min after 5% PVI was injected into the conjunctival sac [48]. Therefore, the clinical use of 5% PVP-I in ocular disinfection may pose a risk of causing discomfort to patients’ eyes. These findings suggest that although 5% PVP-I is widely recognized as safe for ophthalmic use, caution should still be exercised regarding its potential to induce oxidative stress and related damage, to ensure the health of patients’ eyes.

Safe doses of far-UVC were as effective as 5% PVP-I in killing germs on the rat ocular surface. A 9 mJ/cm^2^ far-UVC exposure inhibited over 90% of viable bacteria, compared to 80.8% inhibition with 5% PVP-I, suggesting superior disinfection efficacy for far-UVC. Importantly, 9 mJ/cm^2^ far-UVC did not induce corneal apoptosis or oxidative stress, whereas 5% PVP-I caused significant levels of both, known contributors to dry eye and irritative syndromes [49]. Workers’ ocular safety was prospectively monitored for 12 months in a room exposed to 222 nm Far-UVC radiation; no acute or long-term health consequences were noted in human subjects [50]. Acute adverse events, including corneal erosion and conjunctival hyperemia, were not detected by slit-lamp exams, nor were chronic adverse events like pterygium or cataracts found. The corneal endothelial cell density, refractive error, and visual acuity throughout the trial did not change. At wavelengths greater than 230 nm, the risk still remains, though, if the filtering device malfunctions or is inadequate. International guidelines (such the 22 mJ/cm^2^ threshold suggested by ACGIH) should be followed while performing safe applications. This suggests that 9 mJ/cm^2^ 222 nm far-UVC may offer a non-irritating and efficient disinfection method for the ocular surface, potentially replacing povidone-iodine in ophthalmic surgery.

## 5. Conclusions

In conclusion, we found that disinfection with 5% PVP-I induces a high level of oxidative stress in corneal tissue. Although high doses of 222 nm far-UVC can cause minor corneal damage, an optimized dose of far-UVC achieves effective bacterial elimination with minimal oxidative stress to the cornea, comparable to that of 5% PVP-I. This study demonstrates the potential of 222 nm far-UVC as a non-irritating preoperative disinfectant in clinical ophthalmology, offering a safer alternative to irritating disinfectants like 5% PVP-I. Furthermore, it highlights the potential of far-UVC as a safe and gentle option for ocular disinfection.

## Data Availability

The data presented in this study are available in the article.
